# *Staphylococcus epidermidis* Antimicrobial δ-Toxin (Phenol-Soluble Modulin-γ) Cooperates with Host Antimicrobial Peptides to Kill Group A *Streptococcus*

**DOI:** 10.1371/journal.pone.0008557

**Published:** 2010-01-05

**Authors:** Anna L. Cogen, Kenshi Yamasaki, Jun Muto, Katheryn M. Sanchez, Laura Crotty Alexander, Jackelyn Tanios, Yuping Lai, Judy E. Kim, Victor Nizet, Richard L. Gallo

**Affiliations:** 1 Division of Dermatology, University of California San Diego, San Diego, California, United States of America; 2 Department of Bioengineering, University of California San Diego, San Diego, California, United States of America; 3 Department of Chemistry and Biochemistry, University of California San Diego, San Diego, California, United States of America; 4 Department of Pediatrics, School of Medicine, University of California San Diego, San Diego, California, United States of America; 5 Skaggs School of Pharmacy and Pharmaceutical Sciences, University of California San Diego, San Diego, California, United States of America; National Institute of Allergy and Infectious Diseases, National Institutes of Health, United States of America

## Abstract

Antimicrobial peptides play an important role in host defense against pathogens. Recently, phenol-soluble modulins (PSMs) from *Staphylococcus epidermidis* (*S. epidermidis*) were shown to interact with lipid membranes, form complexes, and exert antimicrobial activity. Based on the abundance and innocuity of the cutaneous resident *S. epidermidis*, we hypothesized that their PSMs contribute to host defense. Here we show that *S. epidermidis* δ-toxin (PSMγ) is normally present in the epidermis and sparsely in the dermis of human skin using immunohistochemistry. Synthetic δ-toxin interacted with neutrophil extracellular traps (NETs) and colocalized with cathelicidin while also inducing NET formation in human neutrophils. In antimicrobial assays against Group A *Streptococcus* (GAS), δ-toxin cooperated with CRAMP, hBD2, and hBD3. In whole blood, addition of δ-toxin exerted a bacteriostatic effect on GAS, and in NETs, δ-toxin increased their killing capacity against this pathogen. Coimmunoprecipitation and tryptophan spectroscopy demonstrated direct binding of δ-toxin to host antimicrobial peptides LL-37, CRAMP, hBD2, and hBD3. Finally, in a mouse wound model, GAS survival was reduced (along with Mip-2 cytokine levels) when the wounds were pretreated with δ-toxin. Thus, these data suggest that *S. epidermidis*–derived δ-toxin cooperates with the host-derived antimicrobial peptides in the innate immune system to reduce survival of an important human bacterial pathogen.

## Introduction

Antimicrobial peptides (AMPs) are critically important in the host's defense against infections. In particular, cathelicidins are an important class of mammalian AMPs as cathelicidin-deficient mice have enhanced susceptibility to skin and other infections, providing evidence that AMPs are critical to the innate immune defense[1], [2], [3], [4]. AMPs have been shown to be important in multiple cell types, including, but not limited to, macrophages, neutrophils, mast cells, and epithelial cells such as skin keratinocytes. Pathogens come into contact with AMPs via host secretory mechanisms and phagocytosis. In addition, it has recently been suggested that microbes can also be killed by binding to insoluble released nucleic acids, histones and associated with AMPs. This structure can be produced by mast cells or neutrophils, where it is known as neutrophil extracellular traps (NETS) [5], [6]. It has been suggested that NETs entwine and kill circulating pathogens in a web of DNA, histones, and AMPs.

Cathelicidins have multiple functionalities. The peptides provide protection through immuno-modulation, inducing chemotaxis, and through direct pore-formation of a broad range of infectious agents including fungi, viruses, and bacteria. LL-37, a active peptide form of human cathelicidin, is suggested to form an amphipathic α-helix that subsequently inserts into negatively charged membranes forming veritable holes [7]. Previous reports suggest that cellular perforation requires multiple LL-37 peptides which coalesce to form oligomeric complexes [8]. Although complexes have been shown to form, it is not known as to whether LL-37 physically forms heterologous complexes with other antimicrobial α-helical peptides.

*Staphylococcus epidermidis* (*S. epidermidis*), the most prevalent of many cutaneous resident microflora, is generally innocuous. Several studies have identified *S. epidermidis* as a common colonizer of healthy human skin [9], [10] and mouse skin [11]. Much like the gut, the microbiota of the skin is hypothesized to play a mutually beneficial role in the cutaneous niche. We have previously demonstrated that peptides produced by *S. epidermidis* exhibit antimicrobial properties, potentially acting as an additional antimicrobial shield. The physico-chemical properties of the *S. epidermidis* PSM, δ-toxin, is comparable to those properties of the *Staphylococcus aureus* δ-toxin. The peptides are similarly α-helical and form complexes, yet the *S. aureus* δ-toxin, unlike the *S. epidermidis* δ-toxin, lacks antimicrobial activity [12], [13], [14]. The difference antimicrobial activity may be due to the conditions under which activity was assayed or the differences in δ-toxin primary sequence (glutamine at position 3 or the addition of a threonine at position 24 in the *S. aureus* δ-toxin).

Several phenol soluble modulins (PSMs) produced by *S. epidermidis, S. aureus* δ-toxin derivatives, and *Staphylococcus haemolyticus* gonococcal growth inhibitor exhibit antimicrobial properties and activity. The *S. epidermidis* PSMs have also been shown to enhance the antimicrobial activity of LL-37 on Group A *Streptococcus* (GAS)[15].

Previous studies have similarly reported that host AMPs act in synergy to kill bacteria [16]. Specifically, LL-37 and hBD2 have been shown to synergistically kill Group B *Streptococcus in vitro* [17]. Moreover, host AMPs have been shown to act synergistically with an antimicrobial peptide produced by *L. lactis* to inhibit *E. coli* [18]. Thus, we sought to determine whether the antimicrobial δ-toxin, also known as PSMγ, produced by the resident cutaneous microbe, *S. epidermidis*, could interact with the host antimicrobials leading to greater pathogen inhibition and enhancement of the host's innate immune system.

Here, we demonstrate that the antimicrobial PSMs contribute to host innate immunity through interacting with and amplifying the cutaneous antimicrobial response. These data illustrate a novel means by which δ-toxin contributes to the innate immune system, pointing towards a beneficial role for *S. epidermidis* on the skin's surface.

## Results

### δ-Toxin Is Deposited on the Skin and Binds to Neutrophil Extracellular Traps

**Figure 1:**
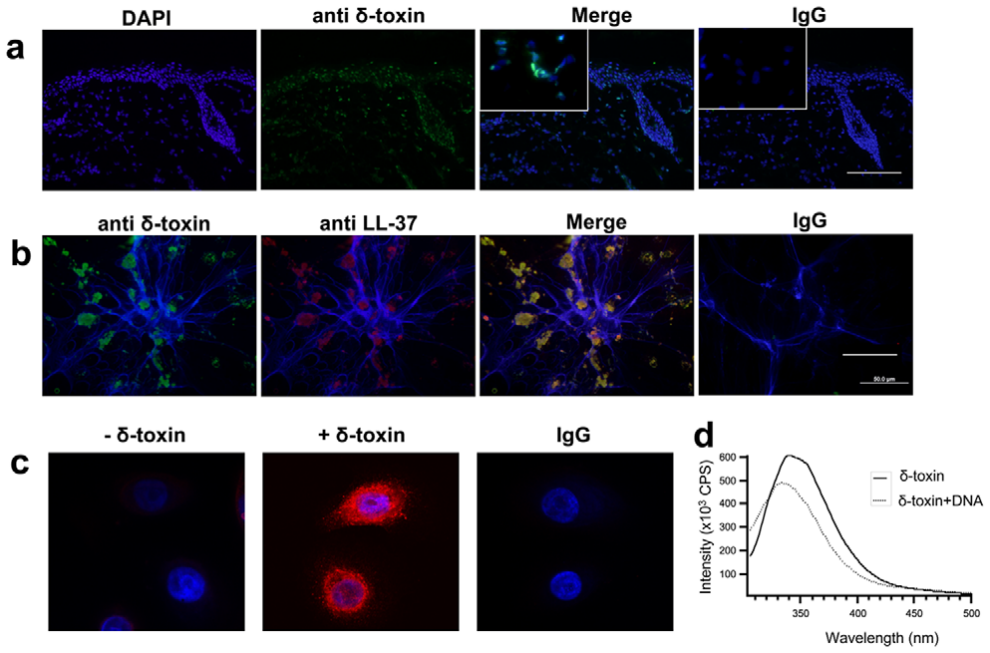
δ-toxin is deposited in the skin by *S. epidermidis* and binds neutrophil extracellular traps. a, normal healthy human skin stained for δ-toxin, showed deposition in the epidermis and dermis. Inset is 40× magnification of δ-toxin in dermis. Bar represents 50 µm. This is a single specimen representative of two. Nuclei are labeled with DAPI (blue) and δ-toxin is labeled with Alexa fluor 488 (green). The far left panel depicts the IgG control for the anti δ-toxin staining. b, δ-toxin was added to neutrophil extracellular traps (NETs), which were subsequently stained for LL-37 and δ-toxin. Staining shows colocalization of antimicrobial peptides along DNA strands. Bar represents 20 µm. Nuclei are labeled with DAPI (blue), δ-toxin is labeled with Alexa fluor 488 (green), and LL-37 is labeled with Alexa fluor 568 (red). The far left panel depicts the IgG control for the anti δ-toxin and anti LL-37 staining. c, primary keratinocytes incubated with (+ δ-toxin) and without (-δ-toxin) δ-toxin then anti δ-toxin staining evaluated. Nuclei are stained with DAPI (blue) and δ-toxin is labeled with Alexa fluor 568 (red). d, tryptophan spectroscopy of δ-toxin in the presence and absence of neutrophil DNA.

**Figure 2:**
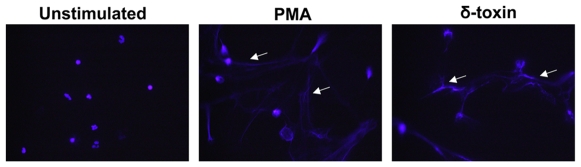
δ-toxin induces NET formation. Freshly isolated human neutrophils were cultured with media only (unstimulated), 25 nM PMA, or 6 µM δ-toxin for 4 hours. Like PMA, δ-toxin induced NET formation as seen by the DNA strands (white arrows).

Phenol soluble modulins are multifunctional and can act to enhance virulence when invasive [19], or as antimicrobials when in direct contact with pathogens such as GAS. To further evaluate the relevance of PSMs as surface antimicrobials on human skin we first determined if δ-toxin was detectable on normal human skin. Immunohistochemistry demonstrated that δ-toxin is abundantly detectable in the normal epidermis, hair follicle and sparsely in the dermis (Figure 1a). Similar staining was observed in a second skin sample from a different individual and staining was confirmed with a second custom-made anti-δ-toxin antibody (data not shown). It is unclear if the two different antibodies recognize different epitopes, as the commercially available epitope is not known. Next, since injured skin rapidly accumulates neutrophils at sites of infection and injury, and these cells act in part to protect the skin through the formation of neutrophil extracellular traps (NETs) containing antimicrobial peptides [6], [20], we evaluated if δ-toxin from surface *S. epidermidis* could interact with NETs and contribute to their activity. δ-toxin was added to PMA-induced neutrophil extracellular traps (NETs) in culture. Addition of δ-toxin to these cells showed that δ-toxin bound to the NETs and colocalized with cathelicidin endogenously released from the neutrophil (Figure 1b). The specifity of the antibody for δ-toxin is demonstrated by lack of staining of normal human keratinocytes in the absence of δ-toxin but positive staining in the presence of δ-toxin (Figure 1c). As the high isoelectric point of this peptide predicts that this association with NETs could occur through DNA binding, δ-toxin association with DNA was next directly evaluated using tryptophan spectroscopy. In buffer alone, δ-toxin's tryptophan emits maximally at 341 nm. In the presence of neutrophil DNA, the maximal emission shifted to 331 nm (Figure 1d). The blue shift caused by the presence of neutrophil DNA suggested a direct association with δ-toxin. Finally, in addition to interacting with NETs, we sought to determine if δ-toxin could also induce formation of NETs *in vitro*. Here, freshly isolated human neutrophils were stimulated with δ-toxin, or PMA as a positive control. Like PMA, δ-toxin was able to induce NETs formation (Figure 2). Thus, these data demonstrate that δ-toxin derived from *S. epidermidis* is deposited on the skin and induces formation of and interacts with NETs.

### δ-Toxin Enhances Endogenous Antimicrobial Activity

**Figure 3:**
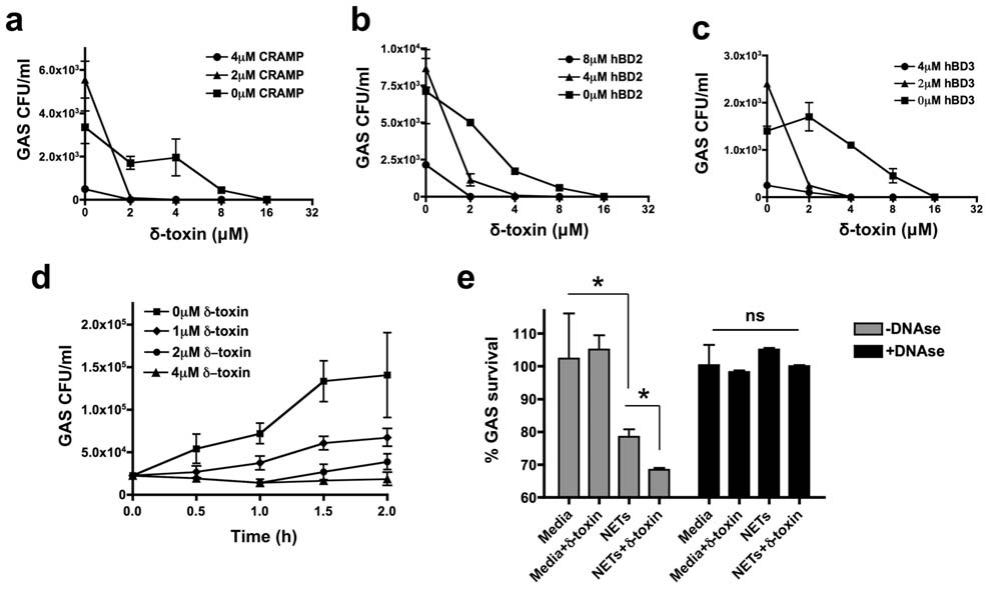
δ-toxin cooperates with host antimicrobial peptides and enhances whole blood and NET killing of GAS. δ-toxin cooperates with host antimicrobial peptides CRAMP (a), hBD2 (b), and hBD3 (c) to kill GAS. d, in whole blood, increasing concentrations of synthetic δ-toxin rendered GAS bacteriostatic. e, δ-toxin added to NETs showed greater GAS killing than NET killing alone. The effect was abrogated by DNase. Data are representative of 2 individual experiments performed in triplicate. *p<0.05. Student's t-test.

To determine whether δ-toxin was able to cooperatively enhance bacterial killing of host AMPs, GAS survival was assessed in the presence of δ-toxin and CRAMP, hBD2, and hBD3. The presence of δ-toxin and low concentrations of host AMPs increased GAS killing in a cooperative manner (Figure 1b and 1c; Figure 3a).

Since δ-toxin enhanced the activity of AMPs, we investigated whether δ-toxin could enhance antimicrobial activity of whole blood. For this, δ-toxin was added to whole blood containing GAS at concentrations below the calculated *in vitro* MIC against this pathogen. Over time, GAS was able to grow in blood without δ-toxin, bacteriostasis was achieved in blood containing 2–4 µM δ-toxin (Figure 3d), As δ-toxin's ability to increase bacterial killing by whole blood may in part be due to the contribution of neutrophil extracellar traps (NETs), and δ-toxin and closely related peptides have been known to lyse red blood cells and neutrophils [19], we next examined if the activity of δ-toxin in the presence of neutrophils is aided by DNA forming the NET. Treatment of NETs and δ-toxin with DNAse eliminated the capacity of both the NETs alone, or NETs with δ-toxin, to inhibit GAS survival (Figure 3e). Hence, the addition of δ-toxin to whole blood or to NETs increases their killing capacity.

### δ-Toxin Physically Binds to Host-Derived AMPs

**Figure 4:**
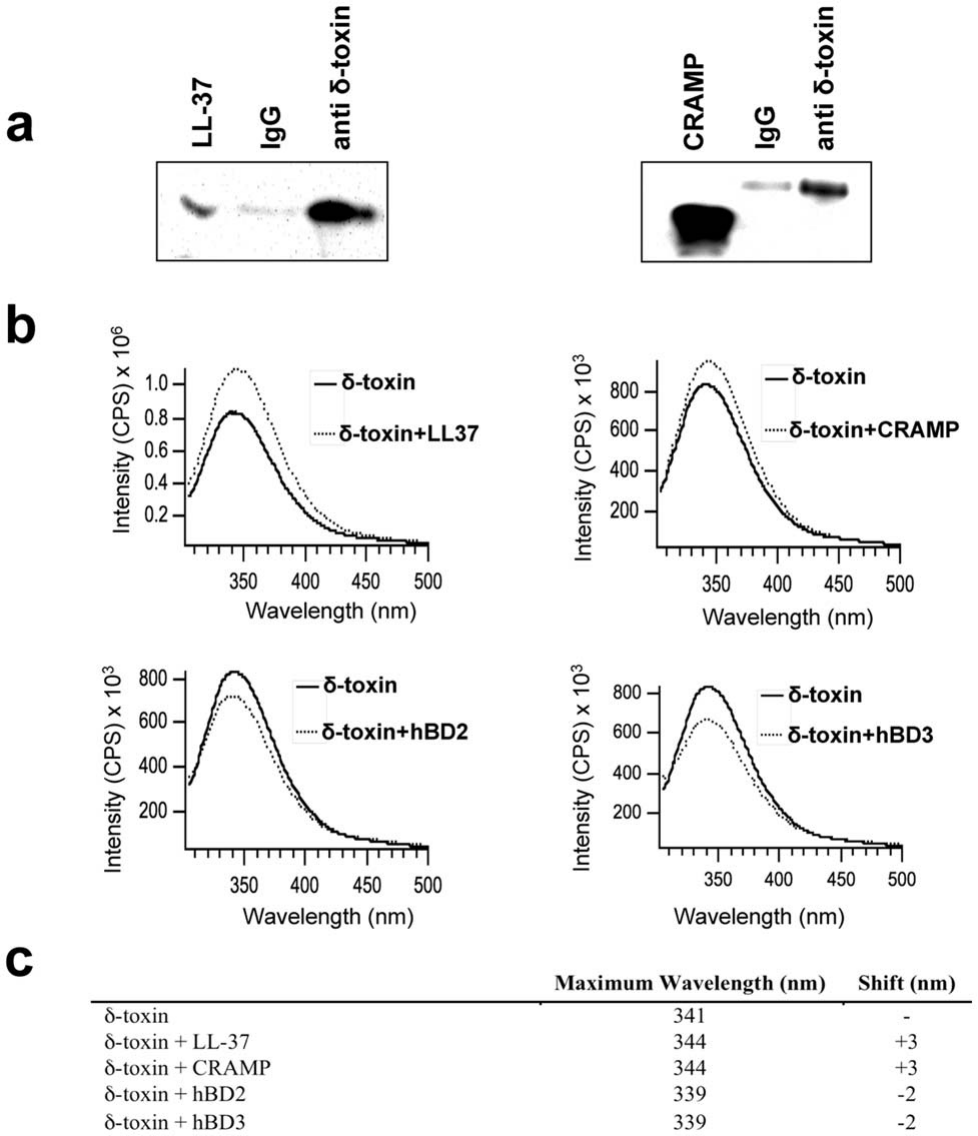
δ-toxin physically binds to host derived antimicrobial peptides. Synthetic 1 µM LL-37 or 1 µM CRAMP was added to *S. epidermidis* supernatants. a, δ-toxin was precipitated and immunoblotted for LL-37 or CRAMP. Immunoblots show co-precipitation of LL-37 or CRAMP with δ-toxin indicating binding. The left and right panels were stained for LL-37 or CRAMP, respectively. The LL-37 or CRAMP positive control standards are shown in the left lane of each panel. The middle and right lanes show relative quantities of LL-37 or CRAMP that precipitated with δ-toxin using anti δ-toxin (right lane) or IgG control to anti δ-toxin (middle lane) b, emission spectra of δ-toxin's tryptophan in buffer or in the presence of LL-37, CRAMP, hBD2, or hBD3. c, table of maximal wavelength emission and shift in wavelength upon addition of host antimicrobial peptide. Data are representative of 2 individual experiments.

Since previous studies suggest that AMPs form heterodimeric and homodimeric complexes [21], and because we found that δ-toxin colocalizes with LL-37 in NETs, we hypothesized that δ-toxin physically binds to the host derived AMPs. In order to determine whether δ-toxin could associate with LL-37 and CRAMP, synthetic host AMPs were added to *S. epidermidis* cell-free, stationary phase supernatant, naturally containing δ-toxin. Precipitation of δ-toxin followed by an immunoblot for LL-37 and CRAMP demonstrated that δ-toxin physically bound to the host derived peptides (Figure 4a).

In order to further confirm interaction between δ-toxin and the host AMPs, tryptophan spectroscopy was performed on δ-toxin in the presence and absence of host AMPs. Unlike δ-toxin, that has a tryptophan at position 15, LL-37, CRAMP, hBD2, and hBD3 are spectroscopically silent because they lack a tryptophan. Due to the spectroscopic silence of the host AMPs, a maximal tryptophan emission shift in δ-toxin during incubation with a host AMP would signify an interaction with and structural change of δ-toxin. To determine the ability of the host AMPs to interact with δ-toxin, the peptide was incubated with host AMPs and tryptophan emission was monitored upon excitation at 290 nm (Figure 4b and 4c). We found that δ-toxin maximal tryptophan emission shifted from 341 nm to 344 nm in the presence of LL-37 and CRAMP, indicating a greater exposure of the tryptophan to the aqueous environment. Conversely, in the presence of hBD2 and hBD3, δ-toxin's maximal tryptophan emission shifted from 341 nm to 339 nm, indicating that the tryptophan resides in a more hydrophobic and embedded environment (Figure 4b and 4c). In addition, we observed that the emission spectra intensity increased upon incubation with LL-37 and CRAMP and decreased upon incubation with hBD2 and hBD3 (Figure 4b). Similarly, we observed that δ-toxin undergoes a conformation change in the presence of genomic keratinocyte DNA. Overall, these data illustrate that δ-toxin directly interacts with, and binds to, host AMPs.

### δ-Toxin Reduces GAS Survival and Inflammation in Mouse Wounds

**Figure 5:**
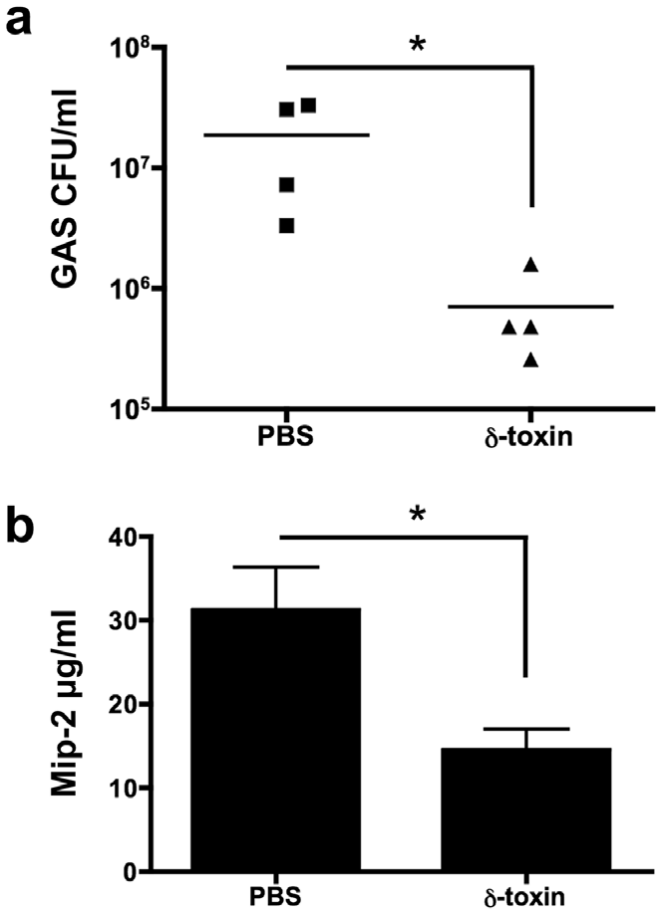
δ-toxin reduces GAS survival and inflammation in mouse wounds. δ-toxin or PBS was added to mouse wounds. After 30 minutes, the treated wounds were challenged with GAS. Wounds were excised, homogenized and plated for GAS CFU/ml (a) or Mip-2 protein levels by ELISA (b). Data are representative of 3 individual experiments performed with n = 3–4. *p<0.05, Student's t-test.

As we have shown that PSMs may contribute to whole blood and neutrophil killing of GAS, we sought to determine if PSMs present in a wound could have a similar protective effect against bacteria, we utilized a mouse wound model. δ-toxin or PBS control was added to 4 mm full-thickness fresh mouse wounds. After only 30 minutes, GAS was added to the wounds to mimic an infected wound. After 18 hours, the infected wounds and surrounding fascia were harvested. GAS survival was significantly decreased in mouse wounds pretreated with δ-toxin but not PBS (Figure 5a). Paralleling the GAS infection, Mip-2 (CXCL2) was significantly decreased in mouse wounds treated with δ-toxin (Figure 5b). These data suggest that δ-toxin reduces GAS survival *in vivo* and may contribute to the innate immune system.

## Discussion

We have previously demonstrated that the PSMδ and PSMγ (δ-toxin) from *S. epidermidis* exhibit antimicrobial activity over pathogens. This activity appeared to result from membrane disruption, a trait common among many antimicrobials [21]. These data support studies suggesting a role of *S. aureus* PSMs in virulence, as the peptides induce pore formation in cells such as neutrophils [19]. Although the PSMs produced by *Staphylococcal sp.* are clearly pore-forming, the impact on the host is likely in part contingent upon the nature of the bacterium and area of residence in the host.

The antimicrobial effect of PSMs on skin pathogens and enhancement of host derived AMPs suggests a role for *S. epidermidis* in the innate immune system of the skin [15]. Other staphylococcal species produce additional or different PSMs that may have other functions or differ in their activity [19]. AMPs have been previously shown to be crucial components of the innate immune system, as illustrated by the susceptibility of *Camp* ^−/−^ mice to GAS [2]. In addition, bacteria have been shown to benefit the human host, as suggested by the hygiene hypothesis and other studies on the gut microflora. Here, we suggest that *S. epidermidis* benefits the host and provides an additional layer of protection against skin pathogens. *S. epidermidis* rather than acting alone, is able to kill pathogens by complementing the host's innate immune system. This close interaction of δ-toxin with the host AMPs insinuates the presence of a mutual relationship between host and bacteria.

We have shown that δ-toxin is deposited in the epidermis and sparsely in the dermis in normal healthy human skin. During scenarios such as injury, δ-toxin may then be able to interact with a variety of cell types including neutrophils. Neutrophils expressing AMPs and forming NETs facilitate eradication of potentially dangerous bacteria [6]. In fact, it has also been shown that injury itself increases AMP production and it is not yet known if injury also induces NETs in circulating neutrophils. As *S. epid*ermidis lives on the skin, injury would bring the bacterium and products in close proximity to the innate immune cells. In this occasion, δ-toxin may increase antimicrobial NET activity and likelihood of pathogen eradication. The NETs illustrate that the host AMPs and δ-toxin intertwine with host DNA. The DNA binding that occurs by AMPs to the NETs may be more than an artifact of charge attraction, but rather suggests a novel role for AMPs. Many studies demonstrate that AMPs cause transcriptional and translational changes in cells. Of course, AMPs may bind receptors, or affect signaling pathways, but these peptides, including PSMs, may directly affect host transcription at the level of DNA binding. Such DNA binding capacity of δ-toxin also suggests a potential role for *S. epidermidis* PSMs in bacterial gene regulation, although *S. aureus* PSMs are not implicated in such [22].

Phenomena of heterologous complex formation and synergy of AMPs from different species have been explored only minimally. Yet AMP synergy has been well documented between AMPs from the same species [16], [17]. Though, one study demonstrated that bacteriocin synergistically enhanced pleurocidin activity against *E. coli* [18]. Although these synergy studies suggest an important relationship between different peptides, a physical interaction between the peptides has not yet been proven. Here, we have shown for the first time, that AMPs from the resident *S. epidermidis* interacts physically with the host AMPs. Co-immunoprecipitation of δ-toxin and CRAMP (or LL-37), along with the tryptophan spectroscopy demonstrated a direct AMP binding. Interestingly, the precipitated CRAMP peptide, ran slightly higher than the synthetic peptide positive control. This may have occurred due to the binding of δ-toxin to CRAMP causing a slower migration.

To show that *S. epidermidis* δ-toxin may contribute to the antimicrobial response *in vivo*, in a scenario in which the peptide enters a wound, we added synthetic δ-toxin to mimic a wound in human skin. Significantly less GAS survived in the mouse wounds that were pretreated with δ-toxin as compared to the vehicle (PBS) control. We also evaluated the levels of a proinflammatory cytokine Mip-2, as a monitor of infection severity. Mip-2, like the number of GAS recovered, indicated an increase in bacterial clearance and general reduction of an inflammatory infection. δ-toxin has been shown to actually induce rather than suppress proinflammatory signaling [23], [24], thus indicating that the reduction in Mip-2 purely parallels the GAS burden. These data suggest that δ-toxin decreases GAS survival in a wound environment. The mechanism responsible for decreased survival of GAS is not clear, but may include neutrophil killing, cooperation with the host AMPs, a surfactant-based effect or activation of the host's innate immune response.

Overall, we show here that δ-toxin is able to bind to host AMPs. Moreover, δ-toxin enhances blood and NET killing of GAS. Finally, the functional reduction of GAS survival in a mouse wound pretreated with δ-toxin suggests a beneficial innate immune role and mutual role for *S. epidermidis*, a common constituent of the skin microbiome.

## Materials and Methods

### Peptides

Formylated δ-toxin was commercially synthesized and purified by HPLC (Quality Controlled Biochemicals, Hopkinton, MA). Mouse cathelicidin-related antimicrobial peptide (CRAMP) and LL-37 were commercially synthesized and purified by HPLC as previously described [25]. Human β-defensins hBD2 and hBD3 were commercially synthesized and HPLC purified (Peptide International).

### In Vitro Antibacterial Studies

Group A *Streptococcus* (GAS) was grown to mid-log phase in Todd-Hewitt Broth (THB, Sigma). A final concentration of 10^5^ CFU/ml were incubated with synthetic δ-toxin at 0, 1, 2, 4, 8, and 16 µM in the presence and absence of LL-37 (0, 4, and 8 µM), CRAMP (0, 2, and 4 µM), hBD2 (0, 2, and 4 µM), and hBD3 (0, 2, and 4 µM). GAS was incubated with peptides in 25% THB and 75% 1x dulbecco phosphate buffered saline (dPBS) for 4 hours at 37°C and then plated on Todd-Hewitt agar for 18 hours before colony enumeration. Graphs are representative of two independent experiments performed in duplicate.

### Whole Blood Assay

Group A *Streptococcus* (GAS) was grown to mid-log phase in Todd-Hewitt Broth (THB, Sigma). A final concentration of 10^5^ CFU/ml was incubated with heparanized human whole blood containing synthetic δ-toxin at 0, 1, 2, or 4 µM. GAS was plated and colonies enumerated at 0.5, 1, 1.5, and 2 hours post inoculation. Graph is representative of two independent experiments performed in triplicate.

### Spectroscopic Measurements

Synthetic δ-toxin (QCB) at 5 µM, was incubated with 5 µM hBD2 (Peptide International), hBD3 (Peptide International), CRAMP, LL-37, or 16 µg/ml genomic neutrophil DNA in 20 mM potassium phosphate buffer, pH 7.3. Fluorescence measurements were taken on a Jobin Yvon SPEX FL3-11 spectrofluorometer equipped with an R928 photomultiplier tube. Conditions for tryptophan fluorescence were as follows: excitation wavelength of 290 nm, emission excitation and emission bandpass of 8 nm and 4 nm, respectively, 1 nm steps, and an integration time of 1s. Spectroscopic curves of host peptide hBD2, hBD3, CRAMP, or LL-37 were subtracted from respective δ-toxin + host peptide curves. Similarly, the spectroscopic curve of DNA was subtracted from the δ-toxin + DNA curve.

### Immunoblot and Precipitation of Antimicrobial Peptides

*Staphylococcus epidermidis* ATCC 12228 was grown for 18 hours to stationary phase in Todd-Hewitt Broth (THB, Sigma). Cells were centrifuged for 5 mins at 5000×g. Supernatants were harvested and filtered through 0.22 µm PES Express Millex GP filter. 1 µM final concentration of synthetic CRAMP or LL-37 was added to 1ml of the filtered supernatant and incubated on nutator at room temperature for 7 hours. Supernatants containing peptides were precleared with 1 µg control IgG antibody and 20 µL Protein A/G-Plus agarose (Santa Cruz Biotechnology) for 1.5 hours at 4°C rotating. Beads were pelleted by centrifugation at 2,500×g for 5 minutes and supernatant was removed and placed in new tube. The 1 ml precleared supernatant was divided into 2 tubes with 500 µL each. To one tube, 1 µg mouse anti-δ-toxin (Abcam) was added. To the other tube 1 µg IgG control was added. Tubes were incubated overnight at 4°C, rotating. 20 µL Protein A/G-Plus agarose were added for 4 hours at 4°C, rotating. Beads were pelleted by centrifugation at 2,500×g for 5 minutes and washed three time in ice cold 1x PBS, spinning down after each wash as described. After the last wash, the supernatant was removed, and beads were resuspended in sample buffer for direct immunoblot. Beads were boiled for 5 minutes and loaded into 16% SDS-PAGE gel along with synthetic peptide as positive control and biotinylated protein ladder (Amersham) and run at 100V for 1.5 hours. Proteins were transferred to polyvinylidene difluoride membrane (Bio-Rad) for 2 hours at 4°C. The membrane was blocked for 1 hour in blocking buffer (PBS, 5% nonfat milk with 3% bovine serum albumin) at room temperature. Incubation in primary antibody rabbit anti-LL-37 at 1∶1000 or rabbit anti-CRAMP at 1∶5000 was performed in blocking buffer overnight at 4°C. Membranes were then incubated in the appropriate horseradish peroxidase-conjugated secondary antibody goat anti-rabbit IgG for 1 hour at 1∶5,000 for 1 hour at room temperature, followed by enhanced chemiluminescence (ECL) detection with the ECL detection kit (Amersham Biosciences).

### Cell Culture and NETs Killing Assay

Neutrophils were purified from healthy donors using the PolymorphPrep TM system (Axis-Shield, Fresnius). 4×10^4^ cells were added to chamber slides in 500 uL RPMI (Biowhitaker). 2×10^5^ cells were added to 96 well plates in RPMI and 5% heat inactivated plasma. NET killing assays were performed as previously described [26], with the exception of adding 6 uM δ-toxin to NETs after 4 hours of PMA stimulation. δ-toxin was allowed to bind to NETs for 1 additional hour, so that the total PMA stimulation time was 5 hours. Media was replaced from NETs, GAS NZ131 was added, spun down for 10 minutes at 800 g and incubated for 30 minutes. GAS was recovered and plated for CFU enumeration.

### Fluorescence Immunohistochemistry of NETs

Freshly isolated neutrophils in chamber slides were incubated with 25 nM PMA in DMEM for 4 hours at standard tissue culture conditions to induce neutrophil extracellular trap formation. Media was carefully removed and replaced with media containing 5 µM δ-toxin for 1 hour. NHEKs, grown on chamber slides, were incubated with 1 µM δ-toxin for 1 hour. Neutrophils and NHEKs were fixed with 4% PFA for 20 minutes at room temperature, washed three time with 1× phosphate-buffered saline (PBS), blocked for 1 hour at room temperature with 3% bovine serum albumin in 1x PBS, and stained with primary antibodies mouse anti-δ-toxin (Abcam), rabbit anti-LL-37, rabbit anti-CRAMP (Quality controlled biochemicals, QCB), rabbit anti-hBD2, and goat anti-hBD3 in 3% bovine serum albumin in 1x PBS overnight at 4°C. After washing the cells 3 times with PBS, the cells were stained with secondary antibodies goat anti-mouse IgG (Sigma), anti-rabbit IgG, and anti-goat IgG in 3% bovine serum albumin in 1x PBS for 1 hour at room temperature. Slides were mounted in ProLong Anti-Fade reagent (Molecular Probes).

### In Vivo Mouse Experiment

The dorsal skin of wild-type C57BL/6 mice, 8–10 weeks old, was removed by shaving and Nair (a depilating agent). 4 mm full thickness punch biopsies were performed on the hair-free dorsal skin. 5 µL PBS or 5 µL 16 µM δ-toxin was added to wounds for 30 minutes. 5 µL of 2×10^6^ CFU/ml was added to treated wounds for an additional 30 minutes. Wounds were harvested using a 6 mm punch biopsy 18 hours post-infection. Wounds were placed in 1 mL PBS with 1 mm zirconia beads. Samples were bead-beated for 1 minute, placed on ice, and bead-beated for an additional minute. Supernatant was serial diluted and plated for CFU on Todd-Hewitt Agar (THA) as well as assayed for Mip-2 levels using ELISA kit following manufacturer's instructions (R&D Systems).
